# Lineage relationships in muscle stem cells: linking functional fate to molecular state

**DOI:** 10.3389/fcell.2026.1930884

**Published:** 2026-09-11

**Authors:** Ohanes Ashekyan, Marie J. Catenacci, Corentin Guilhot, Tony Lin, Michael A. Rudnicki

**Affiliations:** 1 Sprott Centre for Stem Cell Research, Regenerative Medicine Program, Ottawa Hospital Research Institute, Ottawa, ON, Canada; 2 Department of Cellular and Molecular Medicine, Faculty of Medicine, University of Ottawa, Ottawa, ON, Canada; 3 Department of Clinical Science and Translational Medicine, Faculty of Medicine, University of Ottawa, Ottawa, ON, Canada

**Keywords:** continuum, heterogeneity, hierarchical organization, lineage progression, lineage tracing, multi-omics, single-cell, state transitions

## Abstract

Muscle stem cells (MuSCs) represent a subset of satellite cells (SCs) and constitute the stem cell compartment of the skeletal muscle tissue. MuSCs and SCs are quiescent under homeostatic conditions but activate upon injury to drive regeneration and repopulation of the stem cell pool. Heterogeneity has become a central question in MuSC biology, prompting efforts to define MuSC lineage progression through both classical and modern experimental approaches. However, the relationship between the resulting lineage progression models remains unclear. Classical lineage-tracing and transplantation studies uncovered functional heterogeneity and suggested a hierarchical organization. Next-generation sequencing and single-cell technologies later resolved molecular heterogeneity and suggested a dynamic model of continuous state transitions. These two models are often interpreted independently, and their interrelationship is often overlooked. In this review, we critically evaluate the current evidence on the functional and molecular heterogeneity of the MuSC compartment during the quiescence-to-activation transition, emphasizing lineage progression models and the contributions of the regenerative niche and return-to-quiescence mechanisms. We specifically address the link between functional fate and molecular state, proposing a lineage progression model in which these concepts are not mutually exclusive, but rather are intertwined depending on the level and specific transition. Recognizing how functional stemness and dynamic molecular state transitions are related should encourage future experimental designs that combine lineage tracing and transplantation with single-cell multi-omics to obtain a holistic view of MuSC function. This is necessary to develop a better understanding of MuSC deficits in disease and aging, and pave the way for the development of therapeutic strategies targeting functional fate and molecular state links comprehensively.

## 1 Introduction

Satellite cells (SCs) were first described by electron microscopy of skeletal muscle tissue, and found that they reside beneath the basal lamina of myofibers, a localization that gave rise to their original name (Mauro, 1961). The molecular genetic basis for studying myogenesis was advanced by (Rudnicki et al., 1993), where it was shown that myogenic transcription factors Myf5 or MyoD are required for skeletal muscle formation. Pax7 was then identified as a nodal transcription factor that is required for SC specification and maintenance, with Pax7-deficient mice failing to establish and maintain a SC pool (Seale et al., 2000). Indeed, Pax7 is sufficient to induce myogenic specification when ectopically expressed in non-muscle cells (Seale et al., 2004). Pax7 directly regulates Myf5 (McKinnell et al., 2008), where we discuss the specific mechanism in Section 3.2, while its relationship to MyoD is reciprocal, which brings about a balance that governs myogenic progenitor arrest and differentiation (Olguin et al., 2007).

Somatic stem cells are defined as capable of long-term self-renewal and as multipotent (Tweedell, 2017). Long-term self-renewal of SCs was first demonstrated when it was found that about 10% of satellite cells on a single myofiber transplanted into muscle could populate muscle and replenish the satellite cell pool over multiple rounds of injury (Collins et al., 2005). This was later proved by single-cell transplantation experiments into regenerating muscle followed by clonal tracking through serial transplantations (Sacco et al., 2008). Together, these experiments established that a subset of SCs can engraft, proliferate, self-renew, and sustain multiple waves of regeneration upon repeated injury, confirming long-term self-renewal. Importantly, a subset of SCs in adult muscle is capable of differentiating into brown adipocytes as well as myogenic progenitors (Yin et al., 2013a). Multipotency was demonstrated by both lineage tracing and clonal analysis. This multilineage potential was also observed *in vivo* in response to cold exposure. As discussed in Section 4.1, SC lineage tracing studies find that SC fate is largely restricted to myogenic cells under typical animal-house temperatures (Dumont et al., 2015a). Together, these studies laid the conceptual foundation of SC biology and encouraged further investigations into SC heterogeneity during regeneration.

**FIGURE 1:**
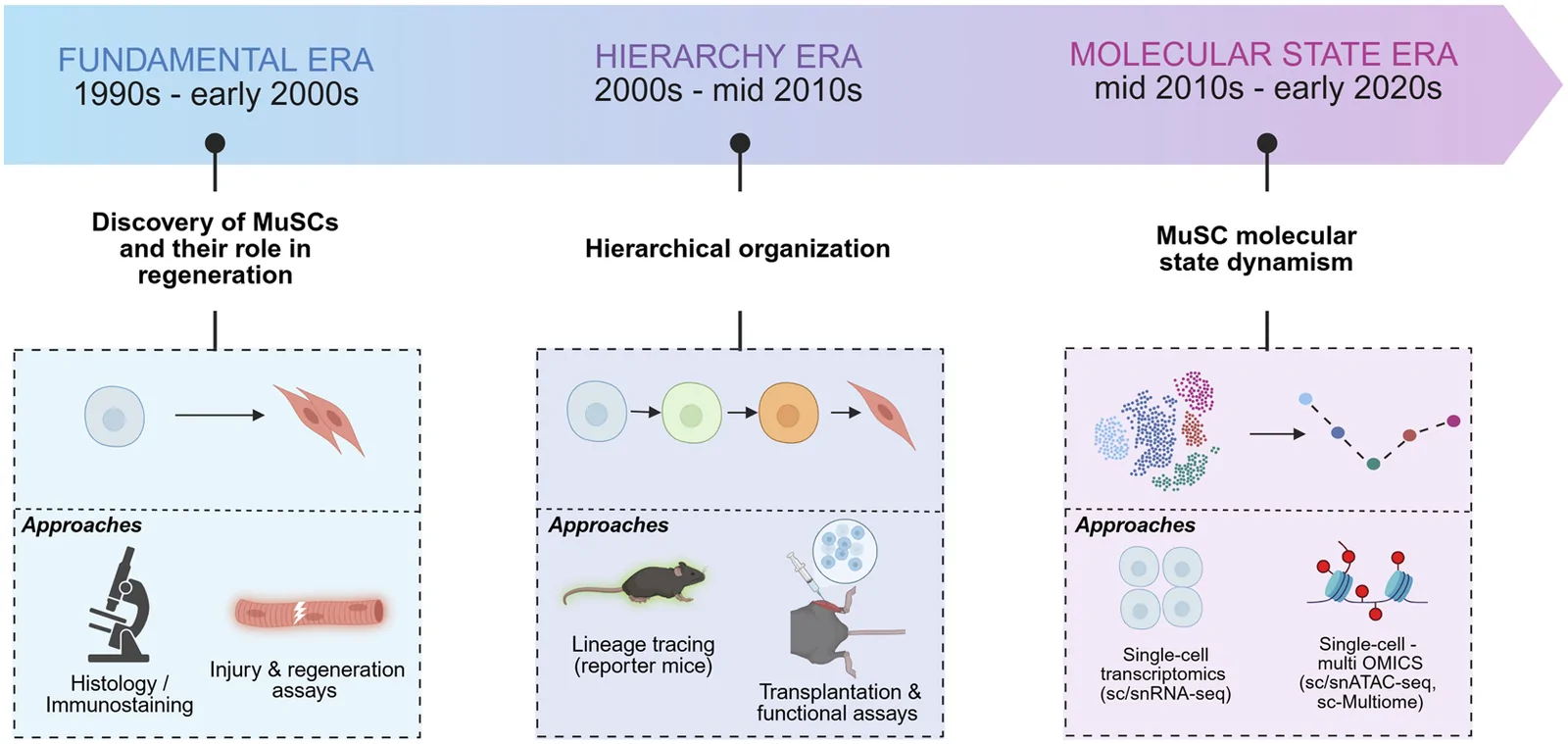
Satellite cell research conceptual historical progression. Experimental and technological advances in satellite cell research allowed the demonstration of different biological aspects of satellite cell biology across the fundamental, hierarchy, and molecular state eras, which are not formally established but represent a conceptual organization.

Genetic reporter models for lineage tracing, complemented by transplantation, revealed that the SC pool varies in regenerative potential, with certain subpopulations showing a greater propensity for long-term self-renewal (Beauchamp et al., 2000; Kuang et al., 2007). These findings established the functional heterogeneity of the SC pool and gave rise to the hierarchical framework. Subsequent advances with next-generation sequencing, particularly at single-cell resolution, made it possible to map the molecular landscape of SC subpopulations across regeneration and revealed the existence of higher-level molecular heterogeneity (van Velthoven et al., 2017; Dell'Orso et al., 2019; De Micheli et al., 2020; Oprescu et al., 2020; McKellar et al., 2021). These approaches also allowed dynamism and reversibility during regeneration to be examined, introducing the concept of molecular state transitions. Thus, we can appreciate the biological knowledge brought by the development of various experimental and technological improvements across different eras in SC research (Figure 1).

Functional and molecular studies address biological questions of a different nature, however, and should not be treated as contradictory; the precise relationship between these two frameworks has yet to be formally established. Throughout this review, we distinguish functional fate from molecular state: state continuity is not proposed to parallel fate reversibility since the two have been assessed by different methods. We further distinguish lineage recording evidence, for example from *Myf5Cre;R-YFP* (Kuang et al., 2007), as being more directly tied to functional fate through transplantation validation than to transcriptional profiles obtained from live reporters and sequencing studies. Therefore, Myf5 expression alone is not considered direct fate evidence, and pseudotime inference is taken as transcriptional progression rather than lineage commitment.

Several review articles have nuanced the hierarchy vs. continuum debate; however, this was rarely the central focus. Saber and coworkers surveyed early scRNA-seq-derived molecular heterogeneity without direct comparison to methodologies that had enabled deciphering the functional heterogeneity of SCs (Saber et al., 2020). Tierney and Sacco gathered evidence for functional heterogeneity from lineage tracing and transplantation studies (Tierney and Sacco, 2016); however, they pre-dated the scRNA-seq era that was the main driver of the continuum model. Relaix and colleagues touched upon topics such as mechanistic insights derived from recent methodologies, cellular heterogeneity in the functional context and SC metabolism dynamics, but these topics have been taken in a broad sense (Relaix et al., 2021). This review lays out the current evidence from functional and molecular SC studies, explicitly framing the hierarchy and continuum models as culminations of different methodologies. These frameworks have usually been taken on independently. Here, we attempt to integrate them across SC regeneration timelines to answer how they are related.

Our aim is to motivate integrative experimental designs that link functional stemness, molecular dynamics, and the regenerative landscape, and address their implications in disease and aging. For the implications, we focus specifically on Duchenne Muscular Dystrophy (DMD) and aging as case study examples since both phenomena are well-studied and characterized in detail, which allowed us to assess our proposed integrative framework. The two additionally represent distinct yet complementary contexts arising from intrinsic and extrinsic defects that are compounded. Other contexts where SC fate and state are perturbed, such as cachexia, denervation and disuse atrophy, could also benefit from the same integrative fate-state framework; however, given the scarce evidence, these represent valuable directions for future application of this framework.

## 2 The classical hierarchical model

The earliest evidence for a hierarchical organization of SCs came from characterizing quiescent SCs on isolated myofibers. Most SCs coexpress Myf5, an early transcriptional regulator of myogenic commitment, together with CD34. The authors speculated that a small Myf5^-^/CD34^-^ subset represented a more primitive stem-like population (Beauchamp et al., 2000). This study introduced the idea that SCs are hierarchically organized at the molecular level.

Hierarchy was formally established through *Myf5-Cre/ROSA26-YFP* lineage tracing, demonstrating that approximately 10% of adult SCs had never expressed Myf5. These cells represent a Pax7^+^/Myf5^-^ satellite stem cell population, distinct from the predominant Pax7^+^/Myf5^+^ committed progenitor compartment (Kuang et al., 2007). Functional transplantation experiments into Pax7^−/−^ muscle further suggested that Myf5^-^ cells efficiently repopulated the satellite cell niche and supported serial rounds of regeneration, whereas Myf5^+^ cells displayed limited self-renewal capacity and underwent precocious differentiation (Kuang et al., 2007). Earlier studies by (Collins et al., 2005; Montarras et al., 2005) had already established the self-sufficiency of the SC pool, showing that freshly isolated SCs were capable of regenerating muscle and reconstituting the SC niche in the absence of alternative progenitor sources. Collectively, these findings anchored the hierarchical model in functional terms by associating stem versus progenitor identity with stable differences in Myf5 expression history. It is important to acknowledge, however, that Myf5 expression itself was later shown to be graded rather than binary, with Myf5^low^ cells sharing features of Myf5^-^ as we discuss in depth in Section 3.1 (Ancel et al., 2024). This is a refinement brought about with the development of specific genetic reporter models that tempers Myf5’s binary classification without negating it. This hierarchy was proposed to depend predominantly, though not exclusively, on asymmetric cell division: upon activation, Pax7^+^/Myf5^-^ SCs were shown to divide along the apico-basal axis, giving rise to a more stem-like basal Pax7^+^/Myf5^-^ and a more committed apical Pax7^+^/Myf5^+^ cell (Kuang et al., 2007; Feige et al., 2018). Importantly, symmetric division modes and the polarity machinery orienting the mitotic spindle are discussed in Section 3.6. In addition, quantitative clonal fate mapping proposing that division mode being context and species-dependent is discussed in Section 5, and niche-mediated contributions to division outcomes are discussed in Sections 3.7 and 6.1. Moreover, complementary evidence from (Shinin et al., 2006) identified a population of label-retaining SCs that undergo selective, non-random segregation of template-DNA strand, consistent with stem-like behavior.

Together, these studies established a model in which SC heterogeneity was interpreted as the product of a cellular hierarchy composed of Pax7^+^/Myf5^-^ stem cells, Pax7^+^/Myf5^+^/MyoD^+^ committed progenitors, and Pax7^-^/myogenin^+^ differentiating myoblasts. Within this framework, lineage progression was considered predominantly unidirectional and governed by the sequential activity of the myogenic regulatory factors Myf5, MyoD, Myogenin, and MRF4 (Rudnicki et al., 1993; Charge and Rudnicki, 2004), and reversion to a stem-like state was not considered a central feature of the model. Consequently, functional heterogeneity within the SC compartment was viewed primarily as an intrinsic property of fixed cell populations with stable rather than dynamic and reversible identities. This hierarchical framework became the dominant paradigm for understanding muscle regeneration and provided the conceptual foundation for subsequent studies investigating SC fate determination, self-renewal, and lineage commitment (Yin et al., 2013b).

## 3 Heterogeneity of MuSCs during quiescence-to-activation transition

**FIGURE 2:**
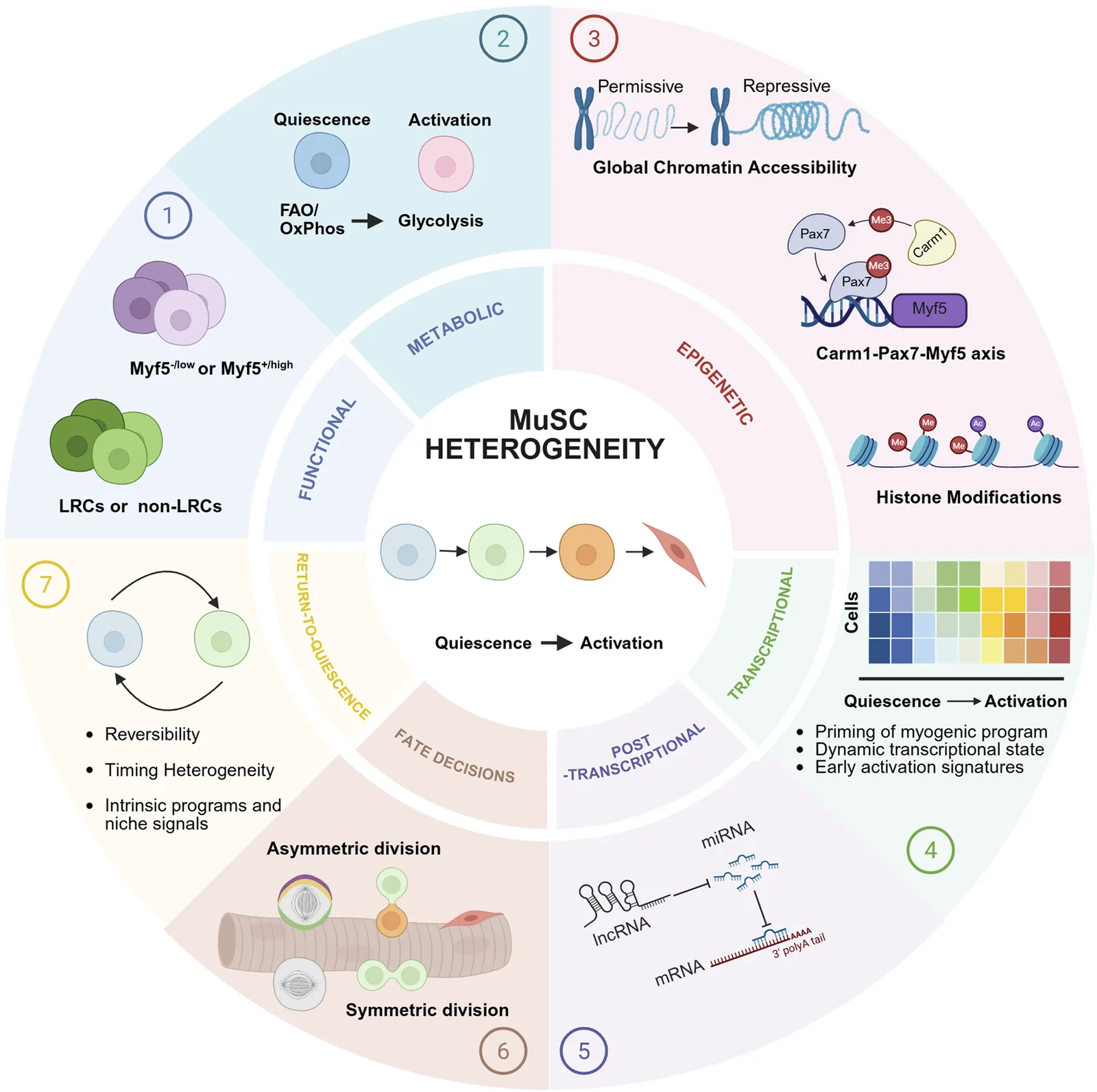
Levels of MuSC heterogeneity during quiescence-to-activation transition. Several independent groups depicted MuSC heterogeneity at multiple classical and emerging levels, namely functional, metabolic, epigenetic, transcriptional, post-transcriptional, fate decisions and return-to-quiescence. These were achieved via differing experimental strategies, increased biological knowledge in certain fields, and advances in next-generation sequencing technology. Some levels of heterogeneity have been studied more robustly than others, which is not reflected by the size of the wheels but the respective section. LRC: label-retaining cell, FAO: fatty-acid oxidation, OxPhos: oxidative phosphorylation, mRNA: messenger RNA, miRNA: micro RNA, lncRNA: long non-coding RNA.

For decades after their discovery, MuSCs were considered a homogeneous population. This view has been continually revised with the development of genetic mouse models, lineage-tracing approaches, and single-cell next-generation sequencing, which together revealed a far more heterogeneous population of cells (Saber et al., 2020). In this section, we discuss the current understanding of MuSC heterogeneity at multiple levels along the quiescence-to-activation transition (Figure 2).

### 3.1 Functional heterogeneity

As previously discussed, the principal stemness distinction among quiescent SCs was made by (Kuang et al., 2007) on the basis of Myf5 expression, with Myf5^-^ MuSCs showing higher stemness and engraftment potential, together with multipotency (Yin et al., 2013a). This central work was supported by recent data identifying two subsets of Myf5-expressing cells, Myf5^low^ and Myf5^high^; Myf5^low^ cells shared several features with the Myf5^-^-subpopulation, including deeper quiescence and greater self-renewal (Ancel et al., 2024). Another study revealed a further layer of heterogeneity by measuring MuSC division rate, distinguishing slow- and fast-cycling subpopulations in adult skeletal muscle. MuSCs were genetically labelled *in vivo* with a TetO-H2B-GFP reporter to track proliferative history through retention of H2B-GFP fluorescence, identifying two distinct subpopulations: label-retaining and non-label-retaining cells. Label-retaining cells (LRCs) underwent three to five rounds of division, whereas non-LRCs performed six or more. The slow-cycling LRC phenotype correlated with deeper quiescence, higher stemness, and a greater contribution to both the Pax7-expressing cells and myonuclei (Chakkalakal et al., 2014). Interestingly, this heterogeneity was absent during embryogenesis, when the microenvironment is globally proliferative (Chakkalakal et al., 2014; Esper et al., 2025b). Together, these observations suggest that functional differences upon activation may be driven, and predicted, by heterogeneity already present in the quiescent MuSC pool.

### 3.2 Metabolic heterogeneity

Although our understanding of MuSC metabolic heterogeneity is far from complete, emerging studies are beginning to address how specific metabolic pathway dependencies shape MuSC quiescence-to-activation transition. Initial work by (Ryall et al., 2015) proposed that MuSC transition to activation is accompanied by a metabolic pathway switch from fatty acid oxidation to glycolysis, pointing to metabolic remodeling during the process rather than simply reflecting increased energy needs. Metabolic pathway transitions were further characterized during MuSC regeneration, showing that quiescent MuSCs rely on fatty acid oxidation-supported mitochondrial oxidative phosphorylation, activated MuSCs on glycolysis, and differentiating myoblasts on oxidative phosphorylation, suggesting that MuSC progression through myogenesis is dependent on distinct metabolic switches (Pala et al., 2018). Furthermore, work by (Baker et al., 2022) presented mitochondrial remodeling as an active regulator of this transition. They showed that OPA1-mediated mitochondrial fusion maintains MuSC deep quiescence, while activation is accompanied by mitochondrial fragmentation. In line with the above, it was suggested that mitochondrial fatty-acid oxidation is crucial for MuSC regeneration and that loss of fatty-acid oxidation impairs MuSC proliferation, highlighting that metabolic flux impacts regenerative capacity (Yue et al., 2025). With the advancements in metabolic profiling, it will be interesting to uncover a more granular metabolic pathway reliance of specific MuSC subpopulations during quiescence-to-activation transition and link metabolic heterogeneity to MuSC fate decisions.

### 3.3 Heterogeneity at the epigenetic level

Another emerging level of heterogeneity in MuSCs lies in the chromatin landscape. Early insights came from global profiling of MuSC epigenetic states in quiescence and activation (Liu et al., 2013). The quiescent MuSC epigenome was largely permissive, dominated by the activating H3K4me3 mark with only a minor repressive H3K27me3 component. Upon activation, H3K4me3 was retained while H3K27me3 increased at transcriptional start sites, producing a more repressive landscape. Single-cell profiling of chromatin accessibility and histone marks should now allow this heterogeneity to be characterized more comprehensively across quiescence and activation. Pax7 itself—the core myogenic transcription factor, expressed at its highest levels in quiescence—activates the myogenic program by associating with the Wdr5–Ash2L–MLL2 histone methyltransferase complex to direct H3K4 trimethylation. Myf5 is a direct Pax7 target, further implicating chromatin dynamics in MuSC heterogeneity (McKinnell et al., 2008). Pax7-mediated induction of Myf5 transcription was later shown to depend on methylation of Pax7 by Carm1, an arginine methyltransferase. Carm1-mediated methylation did not alter Pax7 DNA-binding dynamics but increased H3K4me3 at the promoters of Pax7 target genes. Relevant to heterogeneity, Carm1 binding to Pax7 was inhibited in the non-committed Myf5^-^ compartment and was required to drive Myf5 expression and commitment of myogenic precursors (Kawabe et al., 2012). Carm1’s activity was in turn governed by its phosphorylation status via p38γ/MAPK12 and by its basal-side concentration during asymmetric division, mediated by binding to the dystrophin-glycoprotein complex, which together generated cells with differential Myf5 transcription (Chang et al., 2018). Chromatin dynamics thus impose an additional layer of regulated heterogeneity that acts directly on Pax7 and its targets, most importantly Myf5, to time the quiescence-to-activation transition.

### 3.4 Heterogeneity at the transcriptional level

Several studies have examined MuSC heterogeneity at the transcriptional level, a natural extension of the chromatin-level work, given that transcription depends on chromatin accessibility. Initial single-cell RNAseq using cell expression by linear amplification sequencing (CEL-seq) identified two major transcriptionally distinct subpopulations in quiescent MuSCs. Although the two clusters shared most genes, one had increased expression of immediate early stress-response and heat-shock protein transcripts, suggesting an isolation-induced gene expression signature. These stress-response and heat-shock protein (HSP) gene signatures could be further divided by collagenase incubation time, with HSPs induced later than immediate early genes (van den Brink et al., 2017). A separate study labeled nascent RNA *in vivo* with 4tU across several timepoints (−4 days, −1 day, −6 h), combined with *ex vivo* labeling for 6 h and global profiling of freshly isolated MuSCs. The in-vivo labeled transcriptome harbored various myogenic differentiation transcripts, pointing to an “idling” or “primed” state marked by low overall transcriptional activity that may keep the transcriptional machinery ready for activation. The 6-h–labeled cells were enriched for transcription regulators, whereas 1-day–and 4-day–labeled cells were enriched for metabolism and protein synthesis. The data also indicated a rapid increase in transcription during MuSC isolation—particularly for genes regulating early stress-response, cell cycle, and myogenic commitment—in line with previous reports. Principal component analysis consistently separated the samples, suggesting a range of transitional states across the quiescence-to-activation transition that warrant further study (van Velthoven et al., 2017).

Building on these isolation-induced stress response signatures (Machado et al., 2017), took on an in-situ fixation approach to compare quiescent MuSCs fixed *in situ* with those fixed 3-h and 5-h after isolation. This yielded an accurate quiescence signature of MuSCs and identified early-activation regulators. Principal component analysis revealed clear grouping by timepoint, supporting the idea of transcriptional heterogeneity during the quiescence-to-activation transition. The 3-h and 5-h samples differed from the *in situ*–fixed samples in gene signatures relating to translation, cell cycle regulation and metabolism. As in the nascent-RNA labeling study above (van Velthoven et al., 2017), comparing post-isolation and *in situ*–fixed samples revealed an isolation-induced transcriptional signature, supporting the in-situ fixation approach. Collectively, these studies document the transcriptional heterogeneity of MuSCs during early activation and a differential reliance on specific biological processes. Interest in early-activation transcriptional signatures has been increasing, and several single-cell-based approaches (discussed below) are being utilized to gain insights into activation dynamics.

### 3.5 Heterogeneity at the post-transcriptional level

Beyond transcription, post-transcriptional level regulation of mRNAs involved in this transition adds another layer of heterogeneity through microRNA (miRNA)- and long non-coding RNA (lncRNA)-mediated control of translation. miRNAs inhibit mRNA translation, while lncRNAs can, among other mechanisms, regulate miRNA activity by acting as miRNA sponges (Salmena et al., 2011). Early work showed that miRNAs regulate MuSC proliferation (miR-133) and differentiation (miR-1) (Chen et al., 2006), and that Pax7 is targeted by miR-206, promoting myogenic differentiation (Liu et al., 2012). These findings led to investigations on the role of miRNAs in MuSC quiescence maintenance (Cheung et al., 2012). showed that miRNAs proved essential for actively maintaining MuSC quiescence, with miR-489 acting through the oncogene Dek and declining gradually as MuSCs activate. miR-195 and miR-497 were subsequently shown to maintain quiescence by regulating cell-cycle progression (Sato et al., 2014).

Of interest to non-coding RNAs (ncRNAs) implication in MuSC heterogeneity, a more targeted study on Myf5’s post-transcriptional regulation by (Crist et al., 2012) demonstrated that Myf5 transcripts are sequestered in mRNP granules, inhibited by miR-31. Activatory stimuli were shown to promote the dissociation of these mRNP granules and relief of miR-31 inhibition, suggesting a “primed” state for myogenesis in quiescence. Since 10% of SCs were demonstrated to have never expressed Myf5 by (Kuang et al., 2007), this implies an additional layer of post-transcriptional heterogeneity within the quiescent Myf5^+^ pool. The translational landscape was examined further, revealing a discordance between the transcriptional and translational profiles of quiescent MuSCs: several activation-related transcripts, including MyoD1 and components of the translational and ribosomal machinery, were shown to be translationally repressed (Zeng et al., 2022). Whether these transcripts are differentially regulated by miRNAs and lncRNAs in specific MuSC subpopulations remains to be determined. Especially, single-cell and ncRNA profiling may reveal further post-transcriptional heterogeneity during quiescence-to-activation transition. Consistent with this, miRNA and lncRNA signatures were shown to evolve dynamically from early to late regeneration timepoints after freeze-injury of muscle, strengthening the role for ncRNAs-mediated post-transcriptional heterogeneity (Aguilar et al., 2016).

### 3.6 Heterogeneity during activation and cell fate determination

Upon injury or in disease, MuSCs activate and enter myogenesis, which requires a critical fate decision between self-renewal and differentiation. This decision is governed by the type of MuSC division and its commitment status, primarily defined by Myf5, as described above. Three main division patterns can be distinguished: symmetric Myf5^-^, asymmetric Myf5^-^, and symmetric Myf5^+^, leading to self-renewal by expansion, combined self-renewal and commitment through divergent daughter fates, and committed progenitor expansion, respectively. Asymmetric division is driven by the mitotic spindle orientation and polarity of the cell cortex. Orientation of the mitotic spindle requires partitioning defective protein 3 (Pard3), which triggers a cascade of protein recruitment events that ultimately localizes the nuclear mitotic apparatus (NuMA); NuMA binds to the centrosome and astral microtubules to set the apico-basal orientation of the mitotic spindle (Schober et al., 1999; Siller et al., 2006). Establishment of polarity was demonstrated to be essential for asymmetric division. On the apical side of an asymmetrically dividing MuSC, the multiprotein PAR complex assembles to enforce apicobasal polarity (Guo and Kemphues, 1995; Benton and St Johnston, 2003; Suzuki et al., 2004; Lee et al., 2006; Wirtz-Peitz et al., 2008), while the basal side shows complementary localization of Mark2, which interacts with dystrophin (Dumont et al., 2015b). This drives differential segregation of these proteins and complexes, generating heterogeneous daughter cells. Moreover, other proteins and RNAs also differentially segregate, including NUMB and its ligands, which produce differential NOTCH signaling activity—a pathway crucial for stemness maintenance (Couturier et al., 2012). Although the mechanisms and segregation of polarity proteins are far from fully understood, existing evidence points to an emerging level of heterogeneity during myogenesis that shapes fate outcomes.

### 3.7 Heterogeneity in return to quiescence

MuSC self-renewal is accompanied by a return to quiescence, in which the cells re-enter their homeostatic state and await future regenerative demands. Because MuSC activation is driven by the receptor tyrosine kinase (RTK) ligands FGF and HGF and their downstream signaling cascade, the RTK inhibitor Sprouty1 (Spry1)—already known to be elevated in quiescent MuSCs—was examined in quiescence maintenance (Shea et al., 2010). Spry1 transcript levels were shown to inversely correlate with MuSC activation after injury, then rising again from 12 dpi to reach homeostatic levels by 20 dpi, coinciding with the bulk return of MuSCs to quiescence. Spry1 proved crucial for MuSCs’ return to quiescence: its conditional deletion depleted the self-renewing MuSC pool through apoptosis without affecting differentiation. Although the overall return-to-quiescence landscape was relatively straightforward to map, the specific mechanisms, cellular decisions, and timelines have been difficult to address, given the need for an *in vivo* context. Nevertheless (Cutler et al., 2022), attempted to more closely examine the “return-to-quiescence” dynamics by using an EdU label-retention lineage tracing, with EdU pulses across regeneration timepoints. It was revealed that the majority (∼75%) of MuSCs return to quiescence at 5–10 dpi, and a minority (∼24%) return at 2–4 dpi, whereas at 0–2 dpi, all dividing MuSCs contribute to myofibers—revealing heterogeneity in the timing of quiescence re-acquisition. Interestingly, complementary scRNA-seq data at 4 and 7 dpi revealed a returning cluster of Pax7^high^/MyoD^low^ cells enriched for asymmetric division, self-renewal, and polarity pathways. Moreover, MuSC transplantation into 0- vs. 5-dpi niches yielded an increased number of self-renewing MuSCs in the later niche, suggestive of an extrinsic environmental contribution to the self-renewal/return-to-quiescence balance. Whereas the classical hierarchical model attributes quiescence retention to a strictly cell-intrinsic potential set by early asymmetric division, these findings suggest that the timing of quiescence re-acquisition is heterogeneous and shaped by both intrinsic and extrinsic factors that remain to be defined.

## 4 Resolving MuSC fate and state: Lineage tracing and single-cell sequencing

Cellular fate and state are closely intertwined and tightly correlated in stem cells, particularly during tissue regeneration, and MuSCs are no exception. Classical lineage tracing reveals the differential functional fates of MuSCs across regeneration, while single-cell RNA-seq—by increasing molecular resolution—has led several groups to propose dynamic, transcriptionally distinct MuSC states during myogenesis. In this section, we discuss the current understanding of MuSC functional fate from lineage-tracing strategies, alongside efforts to resolve state-transition dynamics through single-cell approaches that combine multi-timepoint experimental designs and trajectory analysis.

### 4.1 Lineage tracing

Lineage tracing is considered the gold standard for evaluating cell fate decisions *in vivo*, tracking labelled cells across various timepoints (Hsu, 2015). The MuSC field incorporates several variations. Endogenous cells can be labeled with incorporated thymidine analogs such as EdU or BrdU, or transgenic mouse models can be combined with complementary compounds such as doxycycline or tamoxifen to label cells fluorescently (Brack and Rando, 2012). Another common approach is transplantation, where subpopulations of interest are isolated from a defined source, such as a fluorescence reporter mouse or human tissue, and transplanted into a highly controlled environment, typically immunocompromised, irradiated mice. The cells are allowed to engraft and are then analyzed at a chosen timepoint (Feige and Rudnicki, 2020; Hekmatnejad and Rudnicki, 2022). Transplantation of single cells together with serial engraftment studies provides a key approach to clonal analysis of self-renewal potential (Sacco et al., 2008). Lineage tracing was instrumental in demonstrating functional heterogeneity in the MuSC pool across several independent groups. For example (Chakkalakal et al., 2014), used the TetO-H2B-GFP reporter to trace LRC and non-LRC subpopulations during regeneration (Kuang et al., 2007; Ancel et al., 2024). used genetic reporters (Myf5-ROSA26-YFP and dual Pax7/Myf5 reporter, respectively), complemented with transplantation studies for lineage tracing. In order to evaluate the fate of labelled MuSCs, these studies immunostained the myofiber membrane (dystrophin and/or laminin) along with Pax7 to highlight MuSCs.

Most lineage tracing literature suggests two pathways for MuSCs during regeneration. First, MuSCs can undergo differentiation and fuse to myofibers, assessed by quantifying the presence of a label in the myofiber; second, they can self-renew, assessed by colocalization of labels with Pax7 staining (Dumont et al., 2015a). Very few lineage tracing studies find labeled MuSCs in compartments other than the myofiber or MuSC niche after regeneration, indicating that MuSCs are largely restricted to differentiation or self-renewal trajectories, except under extraordinary conditions such as cold exposure that lead to trans-differentiation into brown fat (Yin et al., 2013a). This fate balance between differentiation and self-renewal is essential for proper skeletal muscle regeneration, and occurs relatively early after activation, as (Chakkalakal et al., 2014) showed GFP-labeled cells colocalizing with both Pax7 and the myofiber as early as 7 days post-activation. Future experiments combining barcoding approaches with engraftment of isolated subsets will provide important additional information.

### 4.2 Single-cell sequencing

The earliest timepoint-based scRNA-seq study on MuSCs was conducted by (Dell'Orso et al., 2019) using FACS-isolation of MuSCs from homeostatic muscle, muscle at 60 h post-notexin injury, and cultured primary myoblasts. This approach revealed transcriptional heterogeneity within quiescence, distinguishing close-to-quiescence (cQ) and early-activated (eA) populations according to stress response-related transcripts, with three subpopulations resolved at 60 h post-injury. Aligning these populations along with primary myoblasts along a pseudotime axis revealed a first-of-its-kind cellular transcriptome-based trajectory that enabled the visualization of MuSC progression from quiescence through activation to differentiation. This study addressed global changes across wide regeneration timepoints; later studies by (De Micheli et al., 2020; Oprescu et al., 2020) sampled multiple early-to-late timepoints to capture transient states. De Micheli et al. conducted scRNA-seq on whole hindlimb muscle cell suspensions at 0, 2, 5, and 7 days post-notexin injury; focusing on myogenic populations at D0, D5, and D7 resolved five distinct MuSC and mature skeletal muscle clusters, and trajectory analysis separated quiescent, cycling, and committed states. A parallel study by Oprescu et al. profiled whole-hindlimb populations across regeneration (0, 0.5, 2, 3.5, 5, 10, and 21 days post-cardiotoxin injury), annotating six myogenic clusters—quiescent, immunomyoblast, activated, dividing, committed, and differentiating—whose proportions shifted across timepoints in a manner that trajectory analysis recapitulated as state transitions.

In addition to scRNAseq approaches, other single-cell resolution platforms have also been applied. One such study used single-nucleus ATAC-seq to profile chromatin accessibility in sorted whole-muscle cell populations from 12, 24, 48, 72, and 168 h post BaCl_2_-injury, together with uninjured and contralateral uninjured legs at 6 and 12 hpi. Similarly, this dataset revealed the existence of distinct transient populations and dynamic shifts in cellular epigenomic states over early-to-late regeneration timepoints. Interestingly, pseudotime analysis distinguished differentiation and self-renewal trajectories and inferred a self-renewing cluster (Okafor et al., 2023). Regeneration dynamics have also been modeled computationally (Al-Ghazawi et al., 2025), with differential equation models accurately capturing dynamics for both myogenic and non-myogenic cells. Lastly, other studies (Yartseva et al., 2020; Verma et al., 2024) assessed MuSC state transitions with fewer injury timepoints or in other settings, including development (Xi et al., 2020), Duchenne Muscular Dystrophy (DMD) (Esper et al., 2025a; Esper et al., 2025b; Granet et al., 2025) and aging (Walter et al., 2024).

The majority of the abovementioned studies share a major limitation: they rely on whole-muscle profiling, which captures non-myogenic cells at the expense of resolution within the MuSC compartment. A large-scale scRNA-seq integration study by (McKellar et al., 2021) achieved a more resolved and confident modeling of molecular heterogeneity between intermediate MuSC states along the myogenesis axis, encouraging future experimental designs targeting the MuSC compartment specifically. Together, these efforts move toward a more state-aware understanding of MuSCs across regeneration. Taken with current lineage-tracing work, the growing single-cell characterization of MuSC heterogeneity across regeneration timepoints creates an opportunity to link MuSC fate (from lineage tracing) with MuSC state (from single-cell sequencing). Therefore, studies that directly link fate to state—by combining lineage tracing with single-cell sequencing—are urgently needed. Interestingly, lineage tracing is evolving toward a finer *in vivo* fate resolution (Chen et al., 2025), and combined reporter models such as the dual Pax7/Myf5 model recently applied to MuSC heterogeneity (Ancel et al., 2024) represent encouraging methodological improvements toward the integrated fate-state experimental designs this review calls for.

## 5 Hierarchy and continuum models

MuSC lineage progression has been traditionally explained by the classical hierarchical model and is supported by the phenomenon of asymmetric division, where a MuSC gives rise to a transcriptionally distinct committed daughter cell. However, recent evidence from technological advancements that enabled molecular profiling of individual cells has revealed dynamic state transitions across regeneration, challenging a simple hierarchical model and proposing a model of continuous cell states. Because the evidence for each framework originated from different experimental strategies that addressed different biological questions, in this section, we evaluate them in turn and weigh their respective strengths and limitations.

As discussed above, many studies have proposed that MuSCs are heterogeneous in their regenerative capacity, with sub-populations having a greater propensity for self-renewal (Kuang et al., 2007; Chakkalakal et al., 2014; Ancel et al., 2024). The central studies establishing the hierarchical model relied on lineage tracing and transplantation. Functionally, the hierarchical model accounts well for the durable stemness of MuSCs, the role of asymmetric division in fate transitions, intrinsic differences that partition MuSCs into stem versus progenitor cells, and long-term stemness maintenance by serial regeneration. However, it casts lineage progression as predictable, unidirectional, and irreversible (stem-to-progenitor-to-differentiating cells), whereas single-cell molecular profiling has revealed a more complex, dynamic, and reversible landscape.

Recent NGS-based molecular profiling studies at the single-cell and bulk level, combined with novel algorithms of cell clustering and trajectory inference, suggested a continuous spectrum of MuSC states across regeneration (Machado et al., 2017; van den Brink et al., 2017; van Velthoven et al., 2017; Dell'Orso et al., 2019; De Micheli et al., 2020; Oprescu et al., 2020; Okafor et al., 2023). The continuum model posits partially shared transcriptional signatures, dynamic activation trajectories, and reversible transitions or fate decisions without strict boundaries. It was able to place transcriptional heterogeneity in the context of cellular states, infer gradual activation propensities along with priming, and propose MuSC plasticity with potential return to quiescence. Yet, for all it offers, the continuum model cannot formally establish cell fate associations or prove long-term stemness capacity, which necessitate functional experimental designs.

It is also of equal importance to note the limitations of trajectory analysis beyond the isolation artifacts already discussed (Section 3.4). Pseudotime ordering is based on transcriptional similarity only and is less confident in low-RNA-content quiescent MuSCs (Weinreb et al., 2018). Root and direction are usually chosen from prior knowledge of developmental relationships rather than *de novo*, and cellular organization is dependent on the specific algorithm utilized (Saelens et al., 2019). Moreover, clustering is dependent on user-selected resolution parameters and does not reflect ground truth resolution (Tritschler et al., 2019). These limitations mean that granularity and directionality derived from trajectory inferences should be read as provisional parameter-dependent inferences rather than validated trajectories.

In addition to lineage tracing and single-cell sequencing strategies, quantitative clonal fate mapping offers another strategy to tackle the hierarchy and continuum models. In clonal fate mapping, individual cells are labeled and their clonal progeny are traced over time, which permits inferring division outcomes statistically. In mice (Tierney et al., 2018), showed that SC clonal behavior is dependent on the context. During normal aging, clonal diversity of SCs was preserved, consistent with the notion of a fixed hierarchy; however, with repeated injury, a progressive stochastic clonal drift was observed, leaning more towards a continuum. In zebrafish, live imaging of regeneration with clonal resolution revealed that SCs rely on asymmetric division to self-renew while also generating progenitors, proposing a hierarchical organization (Gurevich et al., 2016). These two clonal studies utilized different methods, species and injury settings, and had different conclusions about SC division mode. This supports this review’s central notion that hierarchy and continuum are not competing and that the homeostatic/regenerative context plays a major role in fate decision.

The disparity between the hierarchical and continuum interpretations arises because the two frameworks rest on different experimental strategies. Hierarchy was emphasized by lineage tracing, while continuum was emphasized by single-cell studies. Lineage tracing tracks fate but not molecular state during regeneration, while single-cell sequencing does the reverse. Because the fate-to-state link has not yet been established using a single experimental strategy, a central open question remains: are MuSCs organized by functional fate, molecular state, or a combination of both? We address this question in Section 6.1.

## 6 Discussion

### 6.1 An integrative model of MuSC fate-state relationships

**FIGURE 3:**
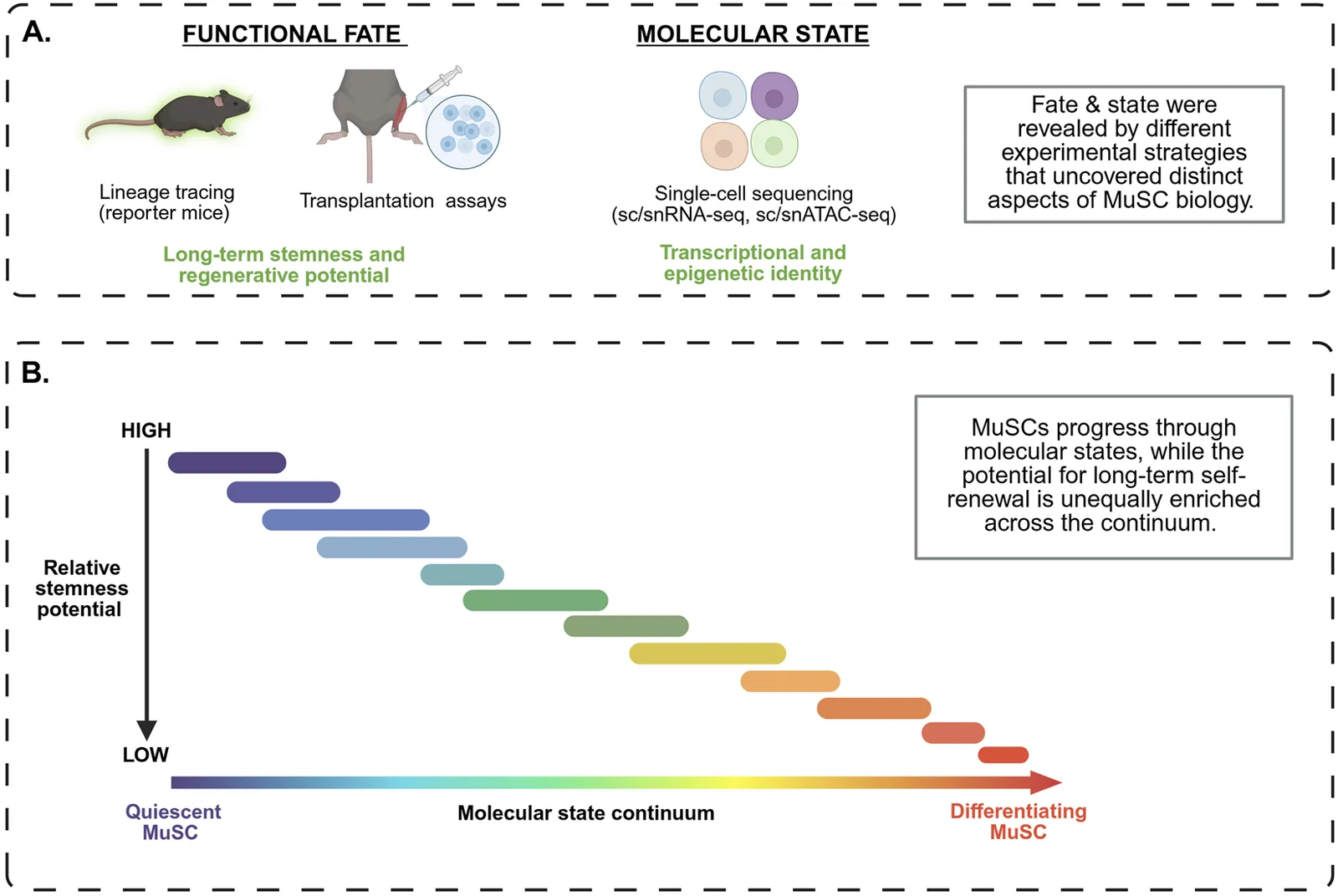
The relationship between functional fate and molecular state. **(A)** Functional fate was uncovered by lineage tracing and transplantation strategies, which established MuSC long-term stemness and regenerative potential. Molecular state was portrayed by single-cell next-generation sequencing approaches, which revealed MuSC transcriptional and epigenetic identity. Each depicted distinct aspects of MuSC biology. **(B)** An integrative view of MuSC progression through regeneration, proposing that functional stemness is embedded within a continuum of molecular states. Y-axis represents the relative stemness potential that is a culmination of lineage tracing and transplantation studies. X-axis represents the molecular state continuum proposed by scRNAseq studies.

In this review, considering the collective evidence in MuSC quiescence-to-activation transition, we conclude that neither a hierarchical nor a continuum-based lineage progression model solely explains the complex myogenesis process. The hierarchical model explains the existence of functionally distinct MuSC subpopulations, whereas the continuum model captures highly dynamic molecular states. These models should not be interpreted as conflicting evidence, as the experimental approaches deciphering both are different in nature (Figure 3A). The two should therefore be treated as complementary rather than conflicting, and future studies combining them would provide the fate-to-state link currently missing from the literature.

With the current state of literature evidence, we propose that an integrative model best captures MuSC lineage progression. Functionally distinct MuSC subpopulations, namely Myf5^-^, Myf5^low^, and label-retaining cells, showed differences in long-term regenerative capacity relative to their counterparts. In conjunction, NGS studies across early-to-late regeneration timepoints, particularly single-cell transcriptomics and epigenomics approaches, revealed molecular state transitions that are not restricted to functionally distinct subpopulations. However, what is almost always overlooked is the relationship between these two observations. The central proposition of this review is that pre-determined functional potential and molecular state should not be interpreted synonymously. Instead, we favour an integrative framework in which functional stemness is embedded within a continuum of molecular states (Figure 3B). The integrative model represents a culmination of intrinsic characteristics of MuSC subpopulations, dynamic molecular states, and regenerative environment context.

The niche contributes at least three extrinsic layers to the integrative model: capillary/endothelial-derived survival signalling (Verma et al., 2024), fibro-adipogenic progenitors (FAPs), which are themselves dynamic during regeneration (Malecova et al., 2018), and transient macrophage-derived proliferative cues (Ratnayake et al., 2021). Niche-related heterogeneity, like the MuSC compartment, is also dynamically state-dependent rather than fixed, which brings about a similar proposal for an in-depth study of the different cellular populations involved during regeneration and their respective implications.

The integrative framework raises several open questions that are actually testable rather than simply proposed. For example, it remains unclear whether functionally defined subsets, such as Myf5^-/low^ or LRCs relate to convergent or distinct locations on the molecular continuum. In addition, whether bi-directional movement along the continuum is possible without the loss of long-term stemness, or instead there are points of no return. Addressing these questions and other questions of similar nature require experimental strategies that enable the capture of fate and state dynamics of individual cells. Examples include heritable barcoding combined with single-cell sequencing, dual Pax7/Myf5 reporters paired with scMultiome profiling, or transplantation of state-sorted MuSC populations.

One unresolved aspect in MuSC lineage progression is the notion of “return-to-quiescence”. Although global return to quiescence was minimally studied by some groups, more efforts should be spent to decipher the precise timing and mechanism involved. With the advent of novel technologies, experimental designs should aim to incorporate genetic reporter models of lineage tracing along with transplantation with single-cell multi-omics. These would permit tracing functionally distinct MuSC-subpopulations’ transition between the proposed dynamic molecular states across regeneration. Importantly, it would enable mapping the potential reversibility of fate decisions, paying particular attention to self-renewal and return to quiescence phenomena. Thus, coupling fate-to-state in controlled regenerative niche environments could help achieve the missing link in true MuSC lineage progression.

A particular illustration of the fate-state relationship proposed above was provided by the “G-Alert” state studies. G-Alert state MuSCs were first found in work that examined the contralateral leg of an initial injury site, where cells had transitioned from deep quiescence (G0) into a primed state. G-Alert functional characteristics include elevated mitochondrial biogenesis, larger cellular size and early cell-cycle entry upon injury. Mechanistically, this was shown to be due to mTORC1 activation downstream of HGF/c-Met signalling. Moreover, transplantation of G-Alert MuSCs showed increased regenerative potential compared to G0 MuSCs (Rodgers et al., 2014). Subsequent work found that G-Alert is induced by hepatocyte growth factor activator (HGFA) systemically upon injury, broadening the concept beyond the neighbouring niche (Rodgers et al., 2017). Further work on upstream regulation of mTORC1 revealed that Gli3 processing at the primary cilium inhibits mTORC1 to maintain MuSCs in the G0 state. Gli3-deficient SCs were demonstrated to enter G-Alert even without injury and harbor an enhanced self-renewal and regenerative capacity (Brun et al., 2022). Thus, G-Alert represents an instance where a specific molecular state has been linked to a functional fate outcome. This offers a template for how future experimental design strategies might resolve the fate-to-state link.

### 6.2 Biological implications in disease and aging

The key question is how the integrative model explains the perturbations seen in disease and aging. Since neither the hierarchical nor the continuum model alone can capture these contexts, given the many contributing factors, both intrinsic and extrinsic, an integrative view would greatly help depict the complete picture.

#### 6.2.1 DMD

The most relevant disease context of skeletal muscle is DMD, caused by genetic loss of dystrophin—a protein required for myofiber structural integrity and signal transduction—which drives continuous cycles of degeneration and regeneration (Emery, 2002; Oak et al., 2003). The dystrophin protein was shown to also be present in MuSCs, supporting a stem-cell-intrinsic component to DMD (Dumont et al., 2015b). Dystrophin loss in MuSCs was shown to impair cell polarity establishment by spatially reorganizing the different players involved, ultimately leading to an abnormal mitotic spindle orientation, resulting in decreased asymmetric division. The consequent imbalance between asymmetric and symmetric division rates results in MuSC hyperplasia accompanied by reduced commitment to the myogenic program (Yamashita et al., 2010; Troy et al., 2012; Dumont et al., 2015b). In conjunction, similar observations were seen in the embryonic context in the *mdx* model during secondary myogenesis with an increased proportion of fetal MuSCs and reduced progenitors (Esper et al., 2025b). This asymmetric-to-symmetric division imbalance was shown to expand the Myf5^-^ MuSC pool at the expense of Myf5^+^ progenitors in the mdx model (Dumont et al., 2015b; Wang et al., 2019). This is specifically a hierarchical framework-based readout that represents one of the several DMD abnormalities described in this section. Although MuSCs could still participate in regeneration, their reduced myogenic potential limits their commitment to fuse and repair myofibers.

**FIGURE 4:**
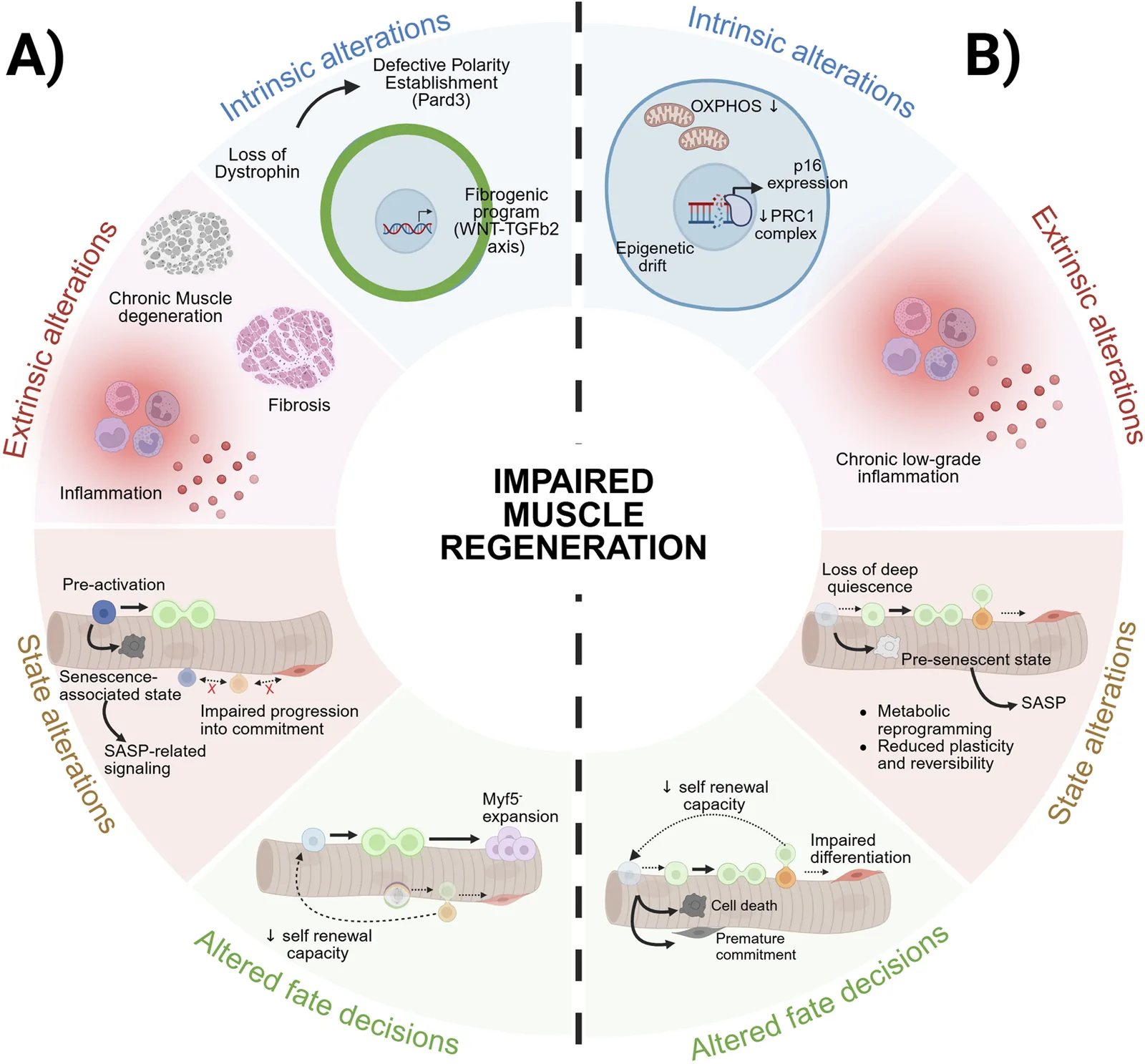
Implication of the integrative framework in DMD and aging. DMD and aging are characterized by specific intrinsic, extrinsic, state, and fate alterations that should be viewed holistically, appreciating the interrelationships between them. This will ultimately lead to a better understanding of these conditions and the development of therapeutics targeting the defects comprehensively. **(A)** DMD. **(B)** Aging.

Another set of evidence from recent studies suggest potential alterations in state dynamics in the DMD context (Esper et al., 2025a). suggested that MuSCs in the *mdx* mouse model have a state of pre-activation despite a lack of commitment. Using scRNA-seq, they denoted pre-activated cellular populations with respective activation-related transcriptional programs that were not present in *wild-type* uninjured muscle. In addition to a pre-activated state, the abnormal polarity, spindle and mitotic issues in DMD-MuSCs were shown to result in a large number of cells that have undergone mitotic arrest, which (Dumont et al., 2015b) predicted would transition to senescence. In line with this (Granet et al., 2025), proposed the emergence of a senescence state via scRNAseq-based characterization of an increased proportion of senescent MuSCs in two separate models recapitulating DMD, namely *mdx* and *D2-mdx*. A small but considerable proportion of DMD-MuSCs were shown to be transitioning into a senescent state, exhibiting a cell cycle arrest signature and higher SA-βGal^+^/Pax7^+^ events. Moreover, in the context of other dystrophies, such as myotonic dystrophy type 1 (DM1), the presence of a senescent subpopulation of myoblasts was shown via scRNAseq, with characteristic senescence-associated secretory phenotype (SASP) production, which promotes a chronic inflammatory and fibrogenic environment (Conte et al., 2023). The SA-βGal^+^/Pax7^+^ and cell-cycle arrest evidence provide the strongest functional support for senescence here, whereas the transcriptional signatures from scRNAseq, including DM1 SASP profile are treated as complementary evidence rather than proof of senescence. Furthermore, work by (Biressi et al., 2014) added that MuSCs trigger an intrinsic fibrogenic program via the Wnt-Tgfβ2 signalling axis, which is characteristic of DMD disease progression. Taken together, these studies indicate that DMD involves not only functional stemness defects but also a restructuring of state heterogeneity, with pre-activated and senescent states expanded at the expense of healthy regenerative states. Thus, perturbations of the DMD phenotype cannot be explained by disruption of either the intrinsic functional stem cell populations or molecular state misregulations alone. Instead, an integration of both, along with the chronically defective regenerative environment, ultimately leads to the alteration of DMD-MuSCs’ regenerative potential (Figure 4A).

#### 6.2.2 Aging

The current state of the literature on the dynamics of MuSCs in aging is heavily contested. Some studies report a decline in MuSC numbers and reduced clonal expansion with age, contributing to impaired regeneration (Day et al., 2010), while others find no change in MuSC numbers (Roth et al., 2000; Brooks et al., 2009). These observed differences are attributed to the nature of the model used in each. An early study by (Chakkalakal et al., 2012) showed aging to be more than just a decrease in MuSC abundance and suggested that aging affects MuSC state regulation, especially quiescence maintenance, where Fgf2 signalling by the aged niche pushes an untimely MuSC quiescence-to-activation transition, leading to gradual depletion. These observations shifted the view of MuSC aging from a mere stem cell depletion phenomenon to a gradual disruption of functional stemness and quiescence maintenance mechanisms, among others.

Increasing efforts were put into deciphering specific regulatory mechanisms governing aged MuSCs quiescence-to-activation transition. Studies taking on the effect of the aged environment on MuSC behaviour showed that several signalling pathways affect MuSC intrinsic characteristics. JAK-STAT signalling was shown to influence MuSC commitment and lead to MuSC pool exhaustion (Price et al., 2014), P38-MAPK signalling was shown to alter asymmetric division and self-renewal dynamics (Bernet et al., 2014; Cosgrove et al., 2014), and a decrease in NOTCH signalling was shown to disrupt quiescence maintenance and lead to precocious differentiation (Bjornson et al., 2012; Mourikis et al., 2012; Liu et al., 2018). In addition to signalling pathway modifications, several publications showed epigenetic alterations, metabolic changes, and loss of transcriptional heterogeneity. Epigenetic repression, mediated by the polycomb-repressive-1 (PRC1) complex on the INK4a locus, was demonstrated to be decreased in aging, leading to precocious expression of p16 in the quiescence-to-activation transition, promoting a pre-senescent state (Sousa-Victor et al., 2014; Zhu et al., 2019). Metabolic dynamics of MuSC quiescence-to-activation transition are also altered with age. The specific changes observed were shown to vary depending on experimental model, tissue, and aging stage (Sartorelli and Ciuffoli, 2025). While young MuSCs shift from fatty-acid oxidation to glycolysis upon activation, aged MuSCs have been shown to rely increasingly on glycolysis in some models, as well as having impaired mitochondrial dynamics and electron transport chain functionality that collectively lead to a more reduced oxidative phosphorylation capacity (Hong et al., 2022). Additionally, MuSCs were shown to exhibit a loss of transcriptional heterogeneity with altered activation-related programs and decreased diversity of populations in aging (Barruet et al., 2023). Moreover, another recent study showed a decrease in “genuine state” MuSCs, described as MuSCs with higher stemness properties in aged MuSCs (Tierney et al., 2018). Together, these findings indicate that aging gradually remodels the regenerative landscape by affecting functional fate transitions and molecular state heterogeneity.

Dynamics of the aging landscape support the integrative framework. A central heterochronic parabiosis study by (Conboy et al., 2005) showed that a young systemic environment rejuvenates aged MuSCs, while an aged environment hinders young MuSCs. While this observation does not solely establish the integrative model, it provides sound evidence that niche and systemic factors also affect MuSC function. This supports the idea that neither functional fate nor molecular state alone is sufficient to explain the dimensions of myogenesis in aging. Considering functional, molecular and niche-level contributions together depicts a more complete picture of MuSC aging (Figure 4B). Aging thus complements DMD in illustrating that neither the hierarchical nor the continuum framework alone is sufficient to understand MuSC dynamics.
